# Efficacy of citronella and ginger essential oil combinations against chicken lice (*Menopon gallinae*) and mites (*Ornithonyssus bursa*): Chemical characterization, contact toxicity, and *in vivo* validation

**DOI:** 10.14202/vetworld.2025.1694-1702

**Published:** 2025-06-26

**Authors:** Nattha Vigad, Prapakorn Tarachai, Sunee Chansakaow, Veerasak Punyapornwithaya, Kridda Chukiatsiri

**Affiliations:** 1 Faculty of Animal Science and Technology, Maejo University, Chiangmai 50290, Thailand; 2 Department of Pharmaceutical Sciences, Faculty of Pharmacy, Chiang Mai University, Chiang Mai 50200, Thailand; 3 Faculty of Veterinary Medicine, Chiang Mai University, Chiang Mai 50100, Thailand

**Keywords:** citronella oil, ectoparasite control, essential oils, ginger oil, *Menopon gallinae*, natural pesticides, *Ornithonyssus bursa*, poultry health

## Abstract

**Background and Aim:**

Ectoparasites, such as *Menopon gallinae* (chicken lice) and *Ornithonyssus bursa* (chicken mites), have a significant impact on poultry health and productivity, resulting in substantial economic losses and potential zoonotic risks. Conventional synthetic pesticides, though effective, pose health and environmental concerns. Hence, plant-based alternatives such as essential oils are gaining attention for their insecticidal properties. This study aimed to characterize the physical and chemical properties of citronella (*Cymbopogon nardus*) and ginger (*Zingiber officinale*) essential oils and to evaluate their acaricidal and insecticidal efficacy, both *in vitro* and *in vivo*, against *M. gallinae* and *O. bursa*.

**Materials and Methods:**

Essential oils were extracted through steam distillation and characterized using specific gravity, refractive index, and optical rotation. Gas chromatography-mass spectrometry (GC-MS) was used to identify major chemical constituents. Contact toxicity assays were conducted on adult lice and mites exposed to essential oil combinations (Citronella [CT]: Ginger [G] in ratios of 70:30, 50:50, and 30:70). *In vivo* trials were performed on naturally infested chickens and mite-contaminated nests, with efficacy assessed at days 1, 7, and 14 post-treatment. Statistical analyses employed general linear mixed models and Tukey’s *post hoc* tests.

**Results:**

GC-MS revealed citral, neral, and limonene as key constituents of citronella oil, and zingiberene and sesquiphellandrene in ginger oil. All essential oil combinations showed 100% *in vitro* mortality of lice and mites by 24 h. *In vivo*, the CT70:G30 formulation achieved the greatest reduction in lice incidence by day 14 (22.67%), followed by trichlorfon (31.33%). For mites, trichlorfon exhibited the highest efficacy (3.33% incidence on day 14), while CT30:G70 also showed notable reduction (40.97%) with no adverse effects observed in treated birds.

**Conclusion:**

Combinations of citronella and ginger essential oils, particularly CT30:G70, offer a promising natural alternative to chemical pesticides for managing poultry ectoparasites. Their high efficacy, rapid action, and safety profile support their use in organic and sustainable poultry farming.

## INTRODUCTION

Poultry parasites contribute to significant eco-nomic losses, costing farmers hundreds of thou-sands of dollars annually [1]. Parasitic infestations pose major challenges in poultry farming, particularly in rural areas and animal farms where chickens are frequently reared. These infestations are broadly categorized into ectoparasites and endoparasites. Ectoparasites, such as lice, mites, fleas, and ticks, are arthropods that infest the skin and feathers of poultry, leading to health problems that reduce productivity and profitability [2]. Although these parasites may not cause immediate mortality in chickens, they can lead to various issues ranging from mild irritation and decreased egg production to severe debilitation and, in extreme cases, death [3]. External parasites are responsible for an estimated $177 million in annual losses [4], severely compromising poultry productivity and farm income. Lice infestations are further classified into sucking lice (*Anoplura*) and chewing lice (*Mallophaga*). Birds infested with lice often develop skin lesions, which can result in secondary bacterial infections and weight loss [5]. Mite infestations may cause severe pruritus, lesions, and even mortality. Moreover, mites contribute to economic losses in poultry due to reduced weight gain, lower egg production, and stunted growth [6]. The northern fowl mite is a permanent ectoparasite that completes its entire lifecycle on the host and can infest chickens irrespective of housing conditions [7, 8]. Thus, external parasite infestations remain a persistent threat to poultry health and productivity.

Various methods have been developed and assessed to control and eliminate these external paras-ites. Conventionally, pesticides such as pyrethroids, organophosphates, carbamates, and macrocyclic lacto-nes have been widely used for the management of ectoparasites in poultry. However, prolonged exposure to these chemicals may result in toxic effects in both animals and humans [9–11]. Furthermore, pesticide use can leave residues in the food chain and the environment, affecting non-target organisms. In response, safer and more sustainable alternatives using natural compounds have been explored. Plant-derived extracts, which are biodegradable and residue-free, offer a safer approach and are increasingly employed for parasite control. Among these, plant essential oils have demonstrated potent insecticidal and repellent activity against various ectoparasites [12]. Citronella essential oil (*Cymbopogon nardus* L.) is rich in terpenoids – secondary metabolites that protect plants from predators, UV radiation, insects, fungi, and bacteria. Their amphipathic properties allow them to interact with cellular membranes, enhancing their bioactivity [13–15]. Citronella oil has demonstrated pharmacological properties including anticonvulsant, analgesic, and anxiolytic effects, as well as efficacy against fungal, bacterial, parasitic, and helminthic inf-estations. Ginger (*Zingiber officinale* Roscoe) is ano-ther medicinal plant; its rhizome is used in both culinary and traditional medicine, exhibiting antioxidant and antidiabetic activities *in vitro* [16]. The insecticidal potential of citronella and ginger oils against lice and mites has also been evaluated *in vivo*. Notably, both oils were found to reduce lice populations over a 14-day period, beginning from day 1 post-application [17].

Despite extensive reliance on synthetic pesti-cides for ectoparasite management in poultry, incre-asing concerns regarding their toxicological risks, environmental persistence, and residual accumulation in poultry products have prompted the search for safer alternatives. Botanical insecticides, particularly essential oils, have shown promising acaricidal and insecticidal properties in laboratory settings. However, several limitations persist in current research. First, most existing studies focus on single essential oils, which often lack broad-spectrum efficacy and prolonged activity due to rapid volatility and instability. Second, the variability in the chemical composition of essential oils, based on plant species, geographical origin, and extraction method, complicates the standardization and reproducibility of results. Third, while *in vitro* bio-assays have demonstrated larvicidal and adulticidal effects of various plant oils, there remains a scarcity of well-structured *in vivo* trials that evaluate their efficacy under realistic farm-like conditions. Specifically, there is limited evidence on the comparative effecti-veness of essential oil combinations, such as citronella (*C. nardus*) and ginger (*Z. officinale*), against multiple poultry ectoparasites, including both *Menopon gallinae* (lice) and *Ornithonyssus bursa* (mites). Furthermore, few studies have comprehensively linked the physic-ochemical properties and chemical constituents of these oils to their observed biological effects, which is critical for establishing quality control benchmarks for future formulations.

The present study aims to address these research gaps by evaluating the insecticidal and acaricidal efficacy of citronella and ginger essential oil combinations against chicken lice (*M. gallinae*) and mites (*O. bursa*), using both *in vitro* contact toxicity assays and *in vivo* trials in naturally infested poultry environments. Specifically, this study seeks to (i) characterize the physical properties (specific gravity, refractive index, and optical rotation) and chemical composition of the individual essential oils using gas chromatography-mass spectrometry (GC-MS); (ii) determine the comparative mortality rates of lice and mites following exposure to different ratios of citronella and ginger oils; and (iii) evaluate the persistence and practical application of these oil combinations under field-like conditions. By integrating physicochemical characterization with biological efficacy assessments, this study provides foundational evidence for the development of plant-based ectoparasiticides, contributing to the advancement of sustainable, residue-free, and environmentally safe strategies for poultry parasite management.

## MATERIALS AND METHODS

### Ethical approval

Ethical approval for this study was obtained from the Animal Ethics Committee, Faculty of Animal Science, Maejo University, Thailand (Approval No. MACUC 024A/2018). All procedures involving animals were conducted in accordance with applicable international, national, and institutional guidelines for the care and use of animals. The research adhered to the ARRIVE guidelines to ensure transparency and rigor in rep-orting *in vivo* experiments, including specific assurances regarding animal welfare, such as the use of humane endpoints and daily monitoring under veterinary supervision.

### Study period and location

The *in vivo* experiments were carried out from October to December 2023, while the *in vitro* experiments were conducted from January to March 2024. This study was conducted in Kalayaniwattana District, Chiang Mai, Thailand.

### Plant essential oils

Two medicinal plant-derived essential oils were used in this study: Citronella (*C. nardus* Rendl), extracted from leaves aged 5–7 months, and ginger (*Z. officinale* Roscoe), extracted from rhizomes aged 4–6 months. Both essential oils were obtained from the Department of Pharmaceutical Sciences, Faculty of Pharmacy, Chiang Mai University, Thailand. The oils were extracted using the steam distillation method and subsequently stored at 4°C under controlled conditions until further analysis.

### Physical characterization of essential oils

#### Specific gravity

The specific gravity of the essential oils was determined following the American Society for Tes-ting and Materials (ASTM) method for liquid fats and oils [18]. The measurement involved comparing the weight of a fixed volume of essential oil to that of an equivalent volume of distilled water at 20°C and 40% relative humidity, using a digital electronic balance.

#### Refractive index

Refractive index measurements were performed using a Japanese-manufactured refractometer (Atago Co., Ltd., Japan). Each sample was analyzed in triplicate, and the mean value was recorded [19].

#### Optical rotation

The optical rotation of essential oils was measured after dilution with analytical-grade chloroform to a concentration of 40 g/L. A CETI Polaris polarimeter (Merck, New Jersey, USA) was used for analysis, conducted in triplicate at 20°C [20].

#### Chemical composition analysis through GC-MS

The chemical constituents of citronella and ginger essential oils were analyzed using an Agilent 7890A gas chromatograph (Agilent Technologies, Santa Clara, CA, USA) coupled with a JEOL AccuTOF-GCv mass spectrometer (JEOL Ltd., Tokyo, Japan), equipped with a DB5-MS capillary column (30 m × 0.25 mm i.d., 0.25 μm film thickness, J and W Scientific, Folsom, CA). The GC oven was programmed to remain isothermal at 40°C for 1 min, then increase at 6°C/min to 250°C, where it was held for 4 min. Helium served as the carrier gas at a flow rate of 1.5 mL/min. The effluent was directed into the mass spectrometry (MS) source through a transfer line maintained at 280°C. Electron impact ionization was employed at 70 eV with a source temperature of 230°C, and mass scanning was performed in the range of 25–800 amu. Compounds were tentatively identified by comparing their mass spectra with entries in the National Institute of Standards and Technology (NIST) MS data library [21].

#### Contact toxicity bioassay (in vitro)

The contact toxicity and persistence of essential oils against adult lice and mites were assessed using a filter-paper bioassay, following the method described in reference [22]. Essential oils were used in a single application before parasite exposure. Solutions were prepared by diluting essential oils in a carrier solvent consisting of distilled water, absolute ethanol, and Tween 20 (50:25:25). This yielded final concentrations of 0.208 g/cm^2^ for lice and 0.416 μg/cm^2^ for mites [17]. Oil mixtures were tested at three citronella-to-ginger ratios: 70:30, 50:50, and 30:70. Trichlorfon served as a positive control for toxicity benchmarking.

Adult lice and mites were collected from infested native chickens and placed in conical tubes at ambient temperature. Samples were transported to the laboratory within 3–4 h to maintain viability. Filter papers (Whatman^™^, Sigma-Aldrich, Missouri, USA) were immersed in the essential oil formulations, dried in a fume hood for 3 min, and placed in petri dishes. Groups of lice (n = 10 per replication, total = 150) and mites (n = 20 per replication, total = 300) were introduced into separate plates and sealed with Parafilm^®^ M (Sigma-Aldrich, Missouri, USA) at room temperature (25°C ± 3°C).

Mortality rates were recorded at 6, 12, and 24 h post-exposure. Each assay was independently repeated 3 times. Mortality was calculated using the formula:

Mortality (%) = (Number of dead insects/Total insects) × 100%

### *In vivo* study

#### Experimental groups

Five treatment groups were established: Three groups received citronella and ginger oil mixtures at ratios of 70:30, 30:70, and 50:50 (denoted CT70:G30, CT30:G70, and CT50:G50, respectively); the fourth group received 0.15% (v/v) trichlorfon as a positive control; and the fifth group served as the negative control and received only the essential oil diluent.

#### Lice infestation trial

A total of 150 lice-infested chickens were sourced from the Faculty of Animal Science and Technology, Maejo University. Birds were divided into five groups (10 chickens per group), maintained under a 12 h light/12 h dark cycle, with *ad libitum* access to feed and water. Environmental conditions were maintained at 28°C–30°C and 40%–50% relative humidity. Birds were housed in individual cages to prevent cross-contamination. Each treatment was replicated 3 times.

At baseline (t = 0), lice were counted within a 2.5 × 2.5 cm^2^ area on each bird. Essential oil formulations (0.208 g/cm^2^, 5 mL/kg body weight) were sprayed once across multiple body regions: Feathers, dorsum, chest, abdomen, flanks, wing undersides, and cloacal area [17]. Lice counts were repeated on days 1, 7, and 14 post-treatment. The incidence percentage was calculated using the formula:

Incidence (%) = (Post-treatment count × 100)/Pre-treatment count

Birds were observed twice daily for 14 days for signs of toxicity (e.g., skin irritation and respiratory distress), in accordance with the Food and Drug Administration guidelines [7, 23]. Observations were carried out by blinded personnel using a standardized checklist.

#### Mite infestation trial

Naturally infested chicken nests in Galyani Vadhana District, Chiang Mai, were included in the study. A total of 15 nests (3 per treatment group) were assigned to the five treatment groups. Essential oil formulations or 0.15% trichlorfon were sprayed directly into the nests. Five sheets of paper were placed in each nest for 5 min to collect mites at baseline (t = 0), and at 1-, 7-, and 14-day post-treatment. Collected mites were immobilized in 5 mL of absolute ethanol before enumeration. Birds from these nests were also observed for any signs of adverse effects for up to 14 days [17].

**Figure 1:**
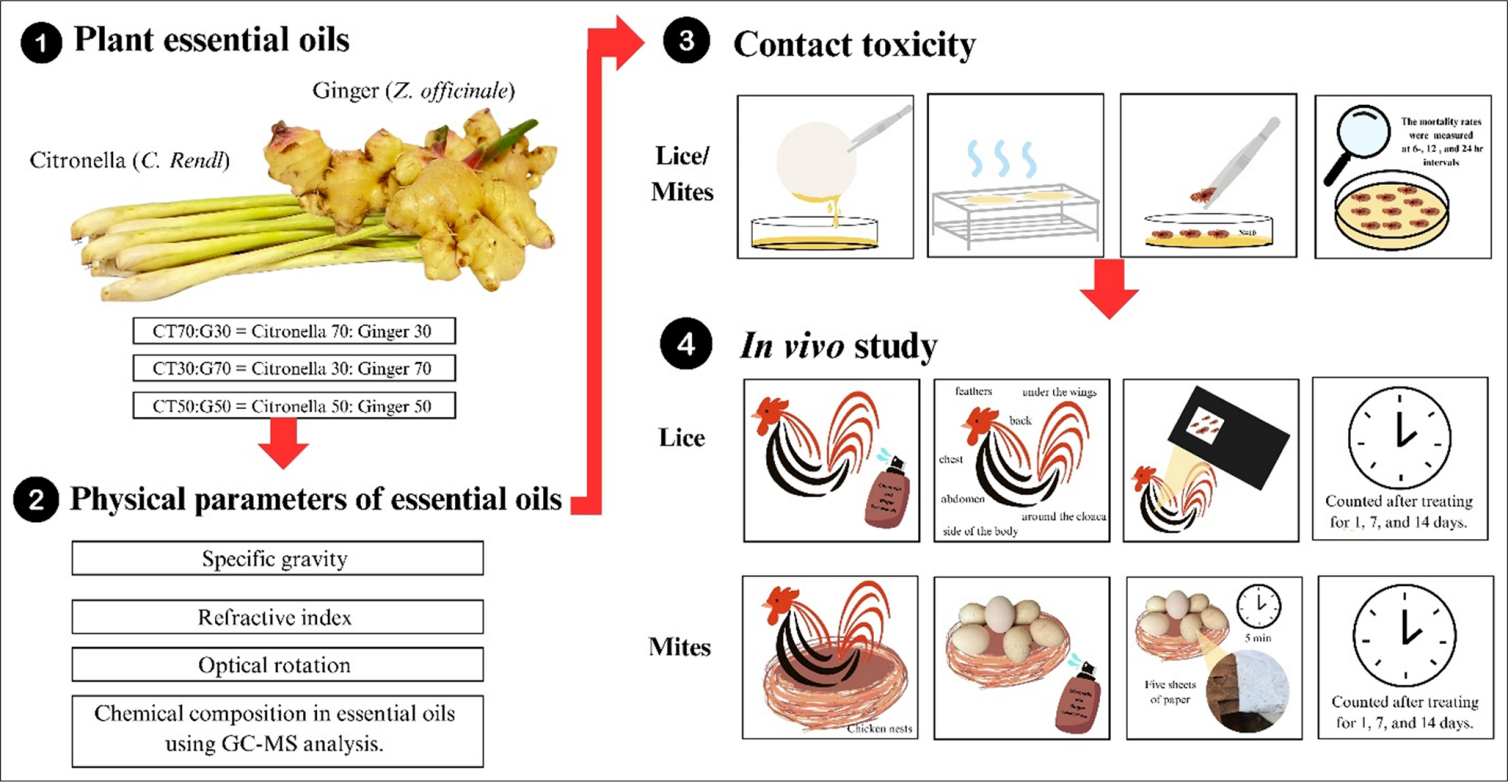
A framework of the study including plant essential oil preparation, determination of physical parameters of essential oil, contact toxicity *(in vitro)*, and in vivo study.

A schematic representation of the experimental framework, including essential oil preparation, physical characterization, and bioassays, is provided in Figure 1.

### Statistical analysis

Data were analyzed using a general linear mixed model (GLMM) appropriate for repeated measures. The model was defined as:

y_ijt_ = μ + tretment_i_ + time_t_ + (treatment * time)_it_ + ε_ijt_

Where y_ijt_ is the j^th^ observation according to the i^th^ treatment at time t, and μ is the overall mean. The term treatment_i_ represents the effect of i^th^ treatment defined as a fixed effect. The term time_t_ denotes a fixed effect due to time. The interaction effect between treatment and time is defined as (treatment*time)_it_. The term ε_ijt_ denotes the error term or model residuals, and it was assumed that ε_ijt_∼NID (0, σ^2^).

Various correlation structures such as first-order autoregressive (AR), compound symmetry, and general symmetry) were evaluated, with model selection based on the lowest Akaike’s Information Criterion (AIC). Assumptions of normality and homogeneity of variance were tested. Tukey’s *post hoc* test was applied for multiple comparisons, and all analyses were performed using SAS University Edition SAS® On Demand for Academics version 9.0 (SAS Institute, Cary, NC, USA).

## RESULTS

### Physicochemical properties of essential oils

The physical characteristics of citronella and ginger essential oils, including specific gravity, refractive index, and optical rotation were systematically analyzed. Citronella oil exhibited a specific gravity of 0.8874, a refractive index of 1.4618, and an optical rotation of +0.34°. In contrast, ginger oil demonstrated a specific gravity of 0.8742, a refractive index of 1.4862, and an optical rotation of −35.52° (Table 1).

**Table 1:** Chemical composition of the essential oils.

Essential oils	Part used	Percentage yield	Chemical composition	Percentage peak area
Citronella oil	Leaf	0.85	Citral	34.23
			Neral	26.71
			Limonene	17.15
Ginger oil	Rhizome	0.92	α-Zingiberene	30.21
			β-Sesquiphellandrene	13.04
			ar-Curcumene	10.47

### Chemical composition by GC-MS analysis

GC-MS analysis identified the major chemical constituents present in both essential oils (Table 1). Citronella oil primarily contained citral, neral, and limonene, while zingiberene was the predominant compound in ginger oil.

### *In vitro* contact toxicity assay

Contact toxicity tests demonstrated the rapid and potent insecticidal activity of citronella-ginger oil combinations against both *M. gallinae* and *O. bursa*. Table 2 presents the percentage mortality of lice expo-sed to various oil formulations over a 12-h period. All combinations tested achieved 100% mortality against both lice and mites within 24 h of exposure. Mortality rates measured at 6, 12, and 24 h revealed statistically significant differences among formulations (p < 0.05).

**Table 2:** Mortality (%) of chicken lice at 6, 12, and 24 h using residue filter paper contact assay.

Treatment^1^	Percentage mortality			SEM	p-value

	Time (h)				

	6	12	24		
CT70: G30	78.02^Bab^	100^Aa^	100^Aa^	3.85	0.0009
CT30: G70	75.06^Bb^	100^Aa^	100^Aa^	4.40	0.0013
CT50: G50	61.11^Bb^	100^Aa^	100^Aa^	7.11	0.005
Control	17.34^c^	18.93^b^	21.59^b^	3.05	0.8809
Trichlorfon	96.36^a^	100^a^	100^a^	0.88	0.1484
SEM	7.49	8.71	8.44		
p-value	< 0.0001	< 0.0001	< 0.0001		

1 CT70: G30=Citronella 70: Ginger 30, CT30: G70=Citronella 30: Ginger 70, CT50: G50=Citronella 50: Ginger 50 All values provided as mean±standard error of triplicate measurements. The lowercase letters in the same row and the uppercase letters in the same column connected by the different letters are significantly different (p < 0.05). SEM=Standard error of the mean

At the 6-h timepoint, the CT30:G70 (citronella 30%: Ginger 70%) formulation resulted in the highest mortality rates for lice and mites, at 78.02% and 76.67%, respectively. All essential oil mixtures showed complete mortality by 24 h, as summarized in Table 3.

**Table 3:** Mortality (%) of chicken mites at 6, 12, and 24 h using residue filter paper contact assay.

Treatment^1^	Percentage mortality			SEM	p-value

	Time (h)				

	6	12	24		
CT70: G30	50.00B^b^	100A^a^	100A^a^	11.47	0.001
CT30: G70	76.67^a^	100^a^	100^a^	6.54	0.0572
CT50: G50	43.33B^b^	100A^a^	100A^a^	12.75	<0.0001
Control	16.67c	23.33^b^	26.67^b^	3.07	0.1012
Trichlorfon	80.00B^a^	100A^a^	100A^a^	5.16	0.0257
SEM	6.59	8.21	7.85		
p-value	< 0.0001	< 0.0001	< 0.0001		

1 CT70: G30=Citronella 70: Ginger 30, CT30: G70=Citronella 30: Ginger 70, CT50: G50=Citronella 50: Ginger 50 All values provided as mean±standard error of triplicate measurements. The lowercase letters in the same row and the uppercase letters in the same column connected by the different letters are significantly different (p < 0.05). SEM=Standard error of the mean

### *In vivo* efficacy study

#### Lice infestation control

The insecticidal efficacy of the essential oil combinations was further assessed *in vivo* using naturally infested chickens. Compared to the control groups, all three essential oil formulations demonstrated a time-dependent reduction in lice incidence from day 1 to day 14. The CT70:G30 formulation (citronella 70%: ginger 30%) was the most effective, reducing lice incidence to 48.59%, 35.03%, and 22.67% on days 1, 7, and 14, respectively. The positive control group (treated with 0.15% trichlorfon) showed lice incidences of 50.62%, 38.85%, and 31.33% at the same timepoints. Although the CT30:G70 group initially showed a decline in lice incidence from 74.5% on day 1 to 66.6% on day 7, an increase was observed by day 14 (67.42%). This variation was not statistically significant (p > 0.05) (Table 4).

**Table 4:** Effects of essential oil preparations on the incidence of chicken lice and mites.

Insects	Days	Percentage incidence^4^				

		CT30: G70^1^	CT50: G50^2^	CT70: G30^3^	Trichlorfon	Control
Lice	0	100.00^a^	100.00^a^	100.00^a^	100.00^a^	100.00
	1	74.5	62.12	48.59	50.62	90.9
	7	66.6	48.75	35.03	38.85	89.27
	14	67.42	41.88	22.67	31.33	87.16
Mites	0	100.00^a^	100.00^a^	100.00^a^	100.00^a^	100.00^a^
	1	44.80	18.31	21.89	15.39	60.43
	7	48.24	54.10	25.96	6.19	139.60
	14	40.97	86.46	68.07	3.33	310.19

1 CT30: G70=Citronella 30: Ginger 70,

2 CT50: 570=Citronella 50: Ginger 50,

3 CT70: 530=Citronella 70: Ginger 30,

4 The percentage incidence of lice and mites on day 0 was normalized to 100%. The lowercase letters in the same column and uppercase letters in the same row connected with different letters indicate significant differences (p < 0.05)

#### Mite infestation control

In the mite infestation model, the positive control group treated with trichlorfon showed the greatest reduction in mite incidence: 15.39% on day 1, 6.19% on day 7, and 3.33% on day 14. Essential oil-treated groups also exhibited low mite incidence on day 1. However, mite counts increased in all groups by day 7. By day 14, the CT30:G70 formulation achieved a secondary red-uction in mite incidence to 40.97%. No statistically significant increase was noted among the other essential oil combinations at this time point (p > 0.05) (Table 4).

## DISCUSSION

### Significance of physical parameters in essential oil characterization

Physical parameters such as refractive index, optical rotation, and specific gravity are essential for defining the specifications and ensuring the conformity of essential oils. The refractive index serves as a qual-itative indicator of oil purity, although it does not directly quantify constituent concentrations. Optical rotation offers a more precise assessment of purity by measuring the optical activity of chiral molecules. Specific gravity reflects the relative density of an oil compared to water and provides insight into its comp-osition and volatility [24].

For instance, ginger oil from West Sumatra, Indonesia, has been reported to possess specific gravity values ranging from 0.75 to 0.91, refractive indices between 1.4851 and 1.4879, and optical rotation values around −30.2° [25]. In comparison, Thai ginger oil demonstrated values of 0.8742, 1.4862, and −35.52°, respectively [17]. Citronella oil has reported refractive index values ranging from 1.47 to 1.49 and optical rotation values between −9.89° and 0.72° [26], while other studies have noted ranges of 1.483–1.489 and −3°–+1°. The primary constituents of *C. nardus* oil include geraniol (35.7%), trans-citral (22.7%), cis-citral (14.2%), geranyl acetate (9.7%), citronellal (5.8%), and citronellol (4.6%) [27].

### Chemical composition and bioactivity of essential oils

In the present study, ginger essential oil was found to contain 30.21% zingiberene. In general, as the concentration of essential oils increases, the relative proportion of volatile substances tends to decrease. Ginger oil is composed of volatile compounds, oleoresins, and enzymes, including proteases, with a total oil content varying between 1% and 3%, depen-ding on cultivation and storage conditions. The major sesquiterpenes include zingiberene and bisabolene. Ginger essential oil exhibits a range of biological activities, including antimicrobial, antiviral, anti-inflammatory, antinociceptive, and antioxidant effects [28–30]. It is predominantly composed of sesquiterpenes (66.7%), followed by monoterpenes (17.3%) and aliphatic compounds (13.6%), with zingiberene accounting for approximately 46.7% of total sesquiterpenes [31].

Citronella oil in this study contained 34.23% citral, a linear monoterpene aldehyde commonly found in over 85% of lemongrass essential oils [32]. Citral demonstrates broad-spectrum biological activity and is effective against various microbial and parasitic pathogens [33, 34]. The combined use of citronella and ginger oils presents advantages over synthetic chemical pesticides, as these natural oils are less likely to cause hypersensitivity reactions in humans, degrade rapidly in the environment, and are readily available.

### Comparative contact toxicity: Essential oils versus trichlorfon

In contact toxicity assays, trichlorfon demonstrated rapid efficacy, achieving a lice mortality rate of 96.36% within 6 h. Essential oil-based treatments produced mortality rates ranging from 61.11% to 78.02% over the same time frame. However, between 12 h and 24 h post-exposure, all essential oil formulations rea*ched comparable or higher efficacy than trichlorfon, indic-ating their potential as effective alternatives.

Trichlorfon, an organophosphate pesticide, exerts its toxic effects by inhibiting acetylcholinesterase but is associated with substantial health risks to both humans and animals due to its high toxicity (lethal dose 50 [LD_50_ > 500 mg/kg]). These results underscore the potential of essential oils as safer and environmentally sustainable insecticidal agents. Previous studies have similarly demonstrated the potent larvicidal and poric-idal activities of compounds like d-limonene from *Zanthoxylum limonella* and trans-anethole from *Illicium verum*, often surpassing the efficacy of conventional agents such as temephos [35–37].

Other research has highlighted the insecticidal potential of combining clove essential oil with cinn-amon or turmeric for chicken lice control [38]. For example, Soonwera *et al*. [39] reported that a combined formulation of *I. verum* and *Cinnamomum verum* in soybean oil achieved 89.5% repellency against cockroaches within 48 h. Similarly, combinations of *Cymbopogon citratus* with *Eucalyptus globulus* resulted in 100% knockdown and mortality in female insects within 1–24 h post-exposure [40]. Given their traditional use in culinary and medicinal contexts, citronella and ginger oils possess a well-established safety profile for both human and environmental applications.

### *In vivo* performance and formulation dynamics

In the *in vivo* trial, the CT30:G70 (citronella 30%: Ginger 70%) formulation reduced lice incidence by 22.67% by day 14, closely matching the effectiveness of trichlorfon. However, in the mite control study, the CT30:G70 formulation initially led to a decrease in mite incidence on day 1, followed by an increase on day 7, and a subsequent decline by day 14.

These fluctuations may be attributed to the volatility and evaporation characteristics of essential oils. In the fragrance industry, essential oils are categorized into top, middle, and base notes based on their volatility and diffusion properties. Ginger oil, a middle-note compound, exhibits slower evaporation and greater stability, while citronella, a top-note oil, dissipates more quickly [41]. The reduced residual activity of essential oils in mite control may also be influenced by environmental factors such as ambient temperature and humidity. Notably, the CT30:G70 group demonstrated a measurable reduction in mite incidence by day 14, highlighting the influence of oil ratio on acaricidal efficacy.

## CONCLUSION

This study provides compelling evidence for the efficacy of citronella (*C. nardus*) and ginger (*Z. officinale*) essential oil combinations as natural alternatives to conventional chemical pesticides for the control of *M. gallinae* (lice) and *O. bursa* (mites) in poultry. The CT30:G70 formulation (citronella 30%: ginger 70%) exhibited the highest mortality rates in both *in vitro* contact bioassays and *in vivo* evaluations, achieving 100% mortality in laboratory tests and a 22.67% reduction in lice infestation by day 14, comparable to trichlorfon. In mite control, although the effect was less persistent, a notable reduction (40.97%) was observed by day 14 post-application.

Strengths of this study include the dual-phase evaluation (both *in vitro* and *in vivo*), comprehensive physicochemical and chemical characterization using GC-MS, and statistical rigor through GLMM with model selection based on AIC. The integration of chemical profiling with biological efficacy reinforces the scientific basis for using plant-based ectoparasiticides.

From a practical standpoint, these essential oil combinations offer a biodegradable, residue-free, and eco-friendly solution suitable for organic poultry production systems. Their use could reduce dependence on synthetic pesticides, minimize health risks to animals and handlers, and prevent pesticide residues in eggs and meat, thereby enhancing food safety.

However, several limitations should be ackno-wledged. The persistence of efficacy, particularly against mites, was limited under field conditions, likely due to the volatility of the oils. The study also did not evaluate the cost-effectiveness, reapplication intervals, or long-term impact on poultry health and productivity. Environmental variables such as humidity and temperature, which may influence oil stability and efficacy, were not controlled in the field trials.

Future studies should focus on improving the formulation stability (e.g., through nanoemulsions or encapsulation), exploring synergistic effects with other botanical compounds, evaluating different delivery methods (e.g., feed additives and vapor diffusion), and conducting large-scale field trials under commercial poultry farming conditions.

The findings validate the potential of citronella and ginger essential oil blends as sustainable and effective agents for controlling poultry ectoparasites. With further optimization, these formulations could play a transformative role in integrated pest management strategies within the global poultry industry.

## AUTHORS’ CONTRIBUTIONS

NV: Implemented the research, conducted the statistical analysis, and drafted and revised the manuscript. VP: Provided recommendations and assisted in both research writing and statistical anal-ysis. SC: Provided the essential oils and shared her extensive knowledge in chemical component analysis. PT: Designed and conducted the study. KC: Supervised the study and critically revised the manuscript for important intellectual content. All authors have read and approved the final version of the manuscript.
